# Multi-epitope Based Peptide Vaccine Candidate Against *Babesia* Infection From Rhoptry-Associated Protein 1 (RAP-1) Antigen Using Immuno-Informatics: An *In Silico* Approach

**DOI:** 10.1177/11779322241287114

**Published:** 2024-12-16

**Authors:** Samson Anjikwi Malgwi, Victoria T Adeleke, Matthew Adekunle Adeleke, Moses Okpeku

**Affiliations:** 1 Discipline of Genetics, School of Life Sciences, University of KwaZulu-Natal, Durban, South Africa; 2 Discipline of Chemical Engineering, Mangosuthu University of Technology, Durban, South Africa

**Keywords:** *Babesia*, immunoinformatics, *in silico*, live vaccines, multi-epitope, vaccine design

## Abstract

**Objective:**

Babesiosis is a significant haemoparasitic infection caused by apicomplexan parasites of the genus *Babesia*. This infection has continuously threatened cattle farmers owing to its devastating effects on productivity and severe economic implications. Failure to curb the increase of the infection has been attributed to largely ineffective vaccines. This study was designed to develop a potential vaccine candidate.

**Method:**

Rhoptry-associated protein-1 (RAP-1) was used to identify and design a potential multi-epitope vaccine candidate due to its immunogenic properties through an immunoinformatics approach.

**Results and conclusions:**

A multi-epitope vaccine comprising 11 CD8+, 17 CD4+, and 3 B-cell epitopes was constructed using the AAY, GPGPG, and KK linkers. Beta-defensin-3 was added as an adjuvant to potentiate the immune response using the EAAK linker. The designed vaccine was computationally predicted to be antigenic (antigenicity scores: 0.6), soluble (solubility index: 0.730), and non-allergenic. The vaccine construct comprises 595 amino acids with a molecular weight of 64 152 kDa, an instability and aliphatic index of 13.89 and 65.82, which confers stability with a Grand average of hydropathicity (GRAVY) value of 0.122, indicating the hydrophobicity of the construct. Europe has the highest combined class population coverage, with a percentage of 96.07%, while Central America has the lowest population coverage, with a value of 22.94%. The DNA sequence of the vaccine construct was optimized and successfully cloned into a pET-28a (+) plasmid vector. Analysis of binding interactions indicated the stability of the complex when docked with Toll-like receptor-2 (TLR-2). The subunit vaccine construct was predicted to induce and boost sufficient host cellular and humoral responses *in silico.* However, further experimental research and analysis is required to validate the findings.

**Limitation:**

This study is purely computational, and further experimental validation of these findings through *in vivo* and *in vitro* conditions is required.

## Introduction

Babesiosis is regarded globally as one of livestock’s most important tick-borne haemoparasitic infections characterized by an invasion of erythrocytes leading to haemolytic anaemia.^1,2^ It is estimated that tick-borne diseases affect 80% of the cattle population around the globe, especially in the tropics and sub-tropics.^3^ The infection is associated with huge economic importance owing to its clinical manifestation and death in severe cases.^4^ It is a protozoan infection that belongs to the genera *Babesia*; species of importance in cattle are *Babesia bigemina, Babesia bovis*, and *Babesia divergens*.^5^ Ticks act as the natural vectors for transmitting the parasite to the host. However, contaminated needles, blood inoculations, and surgical instruments have been reported to transmit the infection under experimental conditions.^6^ Ticks of the sub-genus *Rhipicephalus* are involved in transmitting the *B bovis* and *B bigemina* infection while *Ixodes* spp transmit *B divergens* infection.^7^ *B bigemina* and *B bovis* are cosmopolitan in tropical and subtropical regions and are regarded as economically important infections in Asia, Africa, Australia, South, and Central America,^8,9^ while *B divergens* are found in some European and North African countries and serve as the main etiological agent of the infection in the continent.^10^ *B divergens* is responsible for zoonotic infection in man, causing mild infections except in immunologically compromised subjects, which could be fatal.^11^ Currently, human activity and climatic changes have been associated with an increase in the number of infections.

The major clinical signs of bovine babesiosis include fever, ataxia, anaemia, and haemoglobinuria (coffee-coloured urine).^12^ The economic impact of bovine babesiosis is associated with quantitative and qualitative reduction of meat and milk yield and significant mortality with consequent implications for food security.^13^ This mortality occurs through foetal loss, which arises from abortion due to fever, usually observed in the first trimester of pregnancy.^14^ It is predominantly a disease of adults, resulting in apparent restriction of animal movement transboundary owing to quarantine laws and further affecting the improvement of cattle breeds.^15^ The additional cost incurred for purchasing drugs and acaricides and the cost of an animal health expert further compounded the economic loss.^16^ Several challenges has been associated with the use of live vaccines,^17,18^ which includes variable protection, virulence reversion, viability, tick transmissibility, limited shell life unless preserved, and marketing and logistic issues associated with vaccine development and production.^5^

The urgent need to develop a reliable, safe, environmentally friendly, and effective vaccine to challenge pathogen evolution and geographical coverage cannot be overemphasized owing to the limitation of available vaccines and acaricidal resistance. *In silico* or reverse vaccinology design has been used to develop vaccines from epitopes through a logical approach explored and utilized against conventional methodologies, which are time-consuming, not cost-effective, and inconsistent selection of antigens.^19^ Several new potential vaccine candidates have been proposed from B- and T-cell epitopes.^20^ Immune epitopes are identified based on computational predictions. The success of this approach relies on antigen identification, careful selection of epitopes that will trigger immunological responses. A major advantage of reverse vaccinology involves using data analysis methods for antigen identification, molecular docking, and test of immunological response through simulation models.^21^ Proteomics has provided access to various tools to analyse protein sequences concerning their function and interaction within the organism.^22,23^ Several proteins have been used to design multi-epitope vaccines (MEVs), such as the spike protein in Feline coronavirus,^24^ rhoptry protein against toxoplasmosis,^25^ and outer membrane protein against anaplasmosis.^26^ *In silico* approaches are very helpful in identifying novel potential vaccine candidates within a shorter duration.

Rhoptry protein is a vital protein present in apicomplexan parasites that plays key role in cell invasion process via their secretion to the membranes.^27,28^ These proteins are an integral part of the apical complex derived from the rhoptry organelles which can generate partial immune response often regarded as antigenic.^29^ These have made them the target antigens in designing vaccines and some vaccine trials in parasites such as *Plasmodium* and *Babesia*. Rhoptry-associated protein-1 (RAP-1) proteins are categorized as being multigenic, and these proteins are polymorphic. The B and T cells may be conserved within strains but not across species.^30-32^ These antigens have been used in diagnostic approaches where serology is employed to detect these antigens, especially in *Babesia bigemina* infections.^33^ The RAP-1 proteins comprise epitopes that play vital roles in inducing humoral immunity in the host, which is highly conserved in many isolates.^34,35^ It has been established that to design an effective vaccine against babesiosis, the cell invasion process must be unlocked as this is an important phase of asexual replication that is immunologically vulnerable. The host immune system targets the merozoites via the humoral components, while antibodies cannot access parasitized erythrocytes.^35,36^

Despite the inability of the RAP-1 recombinant vaccine to produce sufficient protective immunity against virulent *B bovis* infection in experimental infection in calves with no change in the clinical course of the disease.^37^ The antigenic/protective potential of RAP-1 antigen is still generating interest, with recent reports suggesting its ability to induce a long-lasting immune response in vaccinated cattle^38^ and further emphasize the protein as a prospect for designing vaccines against bovine babesiosis.^39,40^ Therefore, this study was designed to identify novel sub-unit vaccine candidates through an immunoinformatics approach where all the pathogens of bovine babesiosis (*B bovis*, *B bigemina*, and *B divergens*) were selected and its validation to explore whether these proteins can induce sufficient immune response through immune simulation. Immunoinformatics serves as an interphase between computer science and experimental methodology; this study will seek to answer questions relating to the validity of the protective potential of the antigen *in silico*.

## Materials and Methods

### Protein sequence selection and filtering

Protein sequences of RAP-1 antigen from the most important species associated with bovine babesiosis namely, *Babesia bovis*, *Babesia bigemina*, and *Babesia bigemina* were searched and identified. Amino acid sequences of these target proteins were obtained from NCBI (National Center for Biotechnology Information) online protein databank (https://www.ncbi.nlm.nih.gov/protein) in FASTA format, with further multiple sequence alignment (MSA) using conserved sequences (CD4^+^ and CD8^+^) to identify sequences that will induce immunological responses. CLUSTAL W (http://www.genome.jp/tools-bin/clustalw)^41^ tool was used for MSA, with all parameters in default mode. Conserved regions that align with the reference sequence and comprise at least 15 amino acids were selected for further evaluation.

### Prediction of antigenicity and topology (transmembrane status)

In designing vaccines, it is vital to determine the antigenicity of the conserved sequences and transmembrane status. A potential vaccine candidate should be able to access the outer membrane and be antigenic. To assess the antigenicity of these sequences Vaxijen v2.0 (http://www.ddg-pharmfac.net/vaxijen/VaxiJen/VaxiJen.html)^42^ was used at a threshold of 0.4. The probable antigenic sequences were identified, and further classification of their transmembrane status using TMHMM v2.0 (http://www.cbs.dtu.dk/services/TMHMM/). As stated earlier, only outer membrane sequences were selected.

### Epitope prediction of T-cells and B-cells

Human HLA alleles will be used for MHC-I and MHC-II binding predictions in this study. These parasites have been reported to induce infection in the human population, especially *B divergens*, which is regarded as an emerging zoonosis.^43,44^ Despite the upsurge in the use of NetMHCpan-4.1 and NetMHC-II pan-4.0 in MHC-I and MHC-II binding predictions, a few limitations have been associated with its use. These include having a limited impact on the performance of epitope prediction, biases in selecting data currently available through previous predictions techniques and biases in failure to share data with T-cell epitope in the mass spectrometry EL data.^45^ Human alleles have been used to develop MEVs in non-human species, such as livestock and poultry.^25^ In cattle, MEVs have been developed using HLA against lumpy skin disease^46^ and bovine leukaemia virus^47^ with proven efficacy *in silico*. It is important to note that the pathogen infects cattle and man. All these factors contributed to the selection of human alleles. Similarly, regarding DNA similarity, the cattle shares 80% of its DNA with humans.^48^

### Cytotoxic T-lymphocytes (CTL/CD8^+^) epitopes and MHC-1 binding alleles

Protein sequences that passed the requirement of transmembrane status tests were submitted to NETCTLv1.2 to predict nonamers able to bind to MHC-I HLA alleles inducing CD8^+^.^49^ The nonamers obtained from the earlier analysis were further submitted to IEDB analysis server (http://tools.iedb.org/mhci/). Stabilized matrix-based method (SMM) was used to analyse the nonamers. The criteria for screening MHC-I binding alleles involved the length of the amino acids peptide, which was 9.0, an IC_50_ value greater than 250 in number, and the use of human alleles as the source of MHC species.^50^ Epitopes obtained after this analysis were analysed for antigenicity at a threshold of 0.5. Sequences that passed the requirement of the threshold set were selected as probable antigenic sequences. Further analysis for MHC-1 immunogenicity with the IEDB tool (http://tools.iedb.org/immunogenicity/). This analysis was conducted to establish epitopes that will trigger sufficient immune response towards any pathogen in an infected host.^51^

### Helper T-lymphocytes (HTL/CD4^+^) epitopes and MHC-II binding alleles

The IEDB MHC-II binding tool (http://tools.iedb.org/mhcii/) was used for predicting the helper T-cell epitopes using the SMM align (stabilization matrix alignment) method with the alleles set at a limit of 15 values for the IC50 was < 250. This approach facilitates the identification of MHC-II HLA alleles that bind effectively with CD4^+^ epitopes. Further analysis of these epitopes with the IFNepitope tool (http://crdd.osdd.net/raghava/ifnepitope/) to identify epitopes capable of inducing cytokine IFN. The support vector machine (SVM) method was utilized for the analysis, and all other parameters were set in default mode. The epitopes were further analysed to identify epitopes that could induce interleukin 4 (IL-4), specifically using the IL4Pred server (http://crdd.osdd.net/raghava/il4pred/). Sequences obtained through these analyses were finally subjected to an antigenicity test.

### Population coverage analysis

The conserved T-cell epitopes comprising 11 CD8^+^ and 17 CD4^+^ were submitted to the Population Coverage online web server (http://tools.iedb.org/population/). The T-cell epitopes and their respective alleles were inputted into the server. The analysis option was set for Class I, Class II, and Class combined. The following regions were selected for the analysis, East Asia, Northeast Asia, South Asia, Southeast Asia, Southwest Asia, Europe, East Africa, West Africa, Central Africa, North Africa, South Africa, West Indies, North America, Central America, South America, and Oceania.

### B-cell epitopes

B-cells play a key role in interacting with B-lymphocytes in vaccines. The epitopes earlier generated using Vaxijen and TMHMM, which fulfilled the requirements of antigenicity and outer membrane status, were used to establish antigens capable of producing antibodies through inducing B-cell response. The ABCpred server (http://osddlinux.osdd.net/raghava/abcpred/)^52^ was used for the analysis. The protocol for the analysis was set at a window length of 16 and a 0.51 threshold. Epitopes conserved (overlapped with CD8^+^) and non-allergenic were finally selected for designing an MEV.

### Conservancy and allergenicity

Epitopes finally generated across all the T-cells and B-cells identified as immunogenic and antigenic were further subjected to the conservancy and allergenicity test. The IEDB tool (http://tools.iedb.org/conservancy/) was used for the conservancy tests across antigens, while the AllerTopv2.0 tool was used to identify sequences based on allergenicity. All allergenic sequences were sorted and discarded, while non-allergenic sequences were selected and used in the final stage of the vaccine design.^53^

### Molecular docking of T-cell epitopes (CTL and HTL)

The resulting CTL and HTL epitopes were docked to HLA-A*68:01 and HLA-DRB1*01:01, respectively. These were most conserved among MHC-I and MHC-II and were bound to most of the epitopes predicted and thus were selected for the docking processes. This approach to docking epitopes has been extensively used in several studies. The structural identities of HLA-A*68:01 and HLA-DRB1*01:01 were retrieved from the RCSB protein bank in a suitable format (PDB) of 6pbh and 1aqd, respectively. Determination of solvent accessibility and flexibility of the binding site was done using Naccess 2.1.1 online package before docking. ATTRACTpep server (accessed on August 10, 2022) was used to conduct the docking process of the epitopes on the ATTRACT server and then the installed ATTRACT software package on the CHPC cluster for South Africa to complete the docking. The lowest energy model for each epitope was selected after the conclusion of the docking process. The Visual Molecular Dynamics (VMD) and Chimera software were used to view the images generated.

### Design of multi-epitope sub-unit vaccines

Epitopes comprising CD8^+^, CD4^+^, and B-cells, having successfully met all the requirements after undergoing various immunoinformatics tests, were used to construct the vaccine. These epitopes were joined to an immunological adjuvant to produce the subunit vaccine construct. A different set of linkers were also utilized to attach the various epitopes generated earlier. The AAY linker was used to join the CD8^+^ T-cells, while the GPGPG linkers were used to attach the CD4^+^ T-cells. The B-cells were joined together using the KK linkers. The EAAK linkers were attached at both ends of the N and C terminals and were also used to attach an appropriate adjuvant at the N-terminal of the sub-unit vaccine. These linkers are vital in ensuring stability, increasing immunogens, producing suitable binding sites, stimulating Helper T-cells, and potentiating epitope presentation.^54^ Beta-defensing-3 (accession no. Q5U7J2) was used as an adjuvant to potentiate the immunogenicity of the MEV.^55^ The UniProt database was used to retrieve the sequence of the adjuvant. Adjuvants are used in vaccines to boost immunologic responses against vaccine antigens.^56^

### Evaluation of multi-epitope sub-unit vaccine

#### Antigenicity, allergenicity, solubility prediction, physicochemical properties, and secondary structure

The multi-epitope vaccine designed was tested for antigenicity at a threshold of 0.5 using the Vaxijen server (http://www.ddgpharmfac.net/vaxijen/VaxiJen/VaxiJen.html). The vaccine’s antigenicity will facilitate its interaction and effective binding with the receptor, especially during the docking phase. A further test of the allergenicity of the MEV using AllerTop v2.0 was done to identify the status of the vaccine in terms of being an allergen or non-allergen. SolPro server (http://scratch.proteomics.ics.uci.edu/) was further used to evaluate the solubility of the vaccine construct, which utilizes the SVM-based tool that predicts the solubility of the protein sequence through a tenfold cross-validation approach with an accuracy of over 74.15%.^57^ Analysis of the physicochemical properties of the MEV was also done using the Protparam 53 server (https://web.expasy.org/cgi-bin/protparam/protparam/) via the expert protein analysis system (EXPASSY). The key indicators of physicochemical properties are the specific number of amino acids, the molecular weight of the MEV, aliphatic index, instability index and hydropathicity GRAVY (Grand average of hydropathicity), to mention a few.^58^ PSIPRED v4.0 (http://bioinf.cs.ucl.ac.uk/psipred/) was used to predict the designed vaccine secondary structure.

### Tertiary structure prediction, refinement, and validation

The tertiary structure of the designed multi-epitope vaccine was predicted using the online Galaxy Web server (http://galaxy.seoklab.org) with the TBM model. Refinement of the predicted tertiary structure of the multi-epitope vaccine was designed using the Galaxy Refine server (http://galaxy.seoklab.org/cgi-bin/submit.cgi?type=REFINE).^59^ The refinement of the predicted structure was done to improve the structural integrity of the MEV. Structural perturbations and relaxations of the MEV predicted five refined models. The refined models produced were further screened based on GDT-H, MolProbity, and root mean square deviation (RMSD) values, and the best-refined model was selected for validation. Validation of the most suitable refined model was done using PROCHECK and ProSA-web online server (https://prosa.services.came.sbg.ac.at/prosa.php), which generated a Ramachandran plot and Z-score, respectively which was used for the final validation and confirmation.^60^

#### Discontinuous (confirmational) B-cell epitopes

Linear and discontinuous B-cell epitopes are described as the determinants of antigenic proteins. It is important to note that higher production of antibodies is associated with confirmational B-cell epitope. Therefore, these cells are considered highly antigenic and the most preferred epitopes, especially in immunogen design.^61^ ElliPro online web server (https://tools.iedb.org/ellipro/) that was used to validate the structure of the three-dimensional (3D) model by predicting B-cell epitopes are also regarded as discontinuous.^62,63^ This interphase produces results for the analysis based on clusters of the residues and the estimation of protrusion index (PI).^64^ The results are interpreted in percentages, which explains the presence of the residues within or outside the ellipsoids representing proteins.^65^

### Protein disulfide bond engineering of the MEV

Disulfide bonds are key in stabilizing a protein’s structural interplay (secondary/tertiary). The refined MEV model was subjected to disulfide engineering using the online server Disulfide by Design v2.12 (https://cptweb.opt.wayne.edu/DbD2). This online server can predict sites of disulfide bond formation.^66^ All options for the analysis, such as intra-chain, inter-chain, and glycine residues, were selected. For disulfide engineering, the χ_3_ angle was set at −87° or +97° ± 10, and C_α_–C_β_–S_γ_ angle was kept at 114.6° ± 10. After the analysis, the create button was used to visualize the 3D structure of the mutant.

#### Immune simulation for MEV efficiency

Analysis of the immune response profiles of the MEV was done using C-IMMSIM (http://kraken.iac.rm.cnr.it/C-IMMSIM/).^67^ This online server evaluates the cellular and humoral response of the designed MEV. Several studies have suggested the use of multiple doses at duration of intervals. A period of at least 4 weeks to 6 months duration has been advised to be used between successive doses.^68,69^ In simulating the immune response of the MEV, three doses will be administered at time steps of 1, 84, and 168 with intervals of 4 and 8 weeks, respectively.^70,71^ All parameters were run in default mode with no lipopolysaccharide (LPS) requirement.

#### Molecular docking of the MEV with Toll-like receptor-2

The effectiveness of a vaccine relies effectively on its binding affinity to a receptor. To evaluate the binding affinity of the MEV vaccine designed, docking of the vaccine was done with a Toll-like receptor-2 (TLR-2) receptor (PDB ID 5D3I) obtained from the protein database (https://www.rcsb.org/). TLR-2 expression is important for the recognition of diverse organisms. It responds to lipoproteins in bacteria, cells of fungal organisms, peptidoglycan, and glycosylphosphatidylinositol (GPI) anchors from parasites.^72^ Several studies have demonstrated the importance of TLR-2 regarding vertebrate immunity.^73^ It has been described as the only TLR to form functional heterodimers with some TLRs. It also has a vast interaction network with many non-TLR molecules, thus facilitating the recognition of a more significant number and variety of pathogen-associated molecular patterns (PAMPs).^74^ This diversity encompasses molecules from different microbial phyla, which include parasites, bacteria, fungi, and viruses. The expression of TLR-2 has been detected in numerous tissues such as endothelial, epithelial, and immune cells.^73,75^ The ubiquitous nature of TLR-2 is consistent with its roles and functions.^73^ Gimenez et al^76^ demonstrated a solid inflammatory and macrophage activation response via the TLR-2-dependent pathway, which ultimately facilitates the control of acute babesiosis and host survival. Similarly, TLR-2 was critical in suppressing *Plasmodium* spp in the pre-erythrocytic stage *in vivo*.^77^ All these factors contributed to the choice of TLR-2 in the study. Before docking, solvent accessibility was calculated for the residues by their water accessibility using the Naccess 2.1.1 package (http://wolf.bms.umist.ac.uk/naccess/) to determine the binding sites for both TLR-2 and MEV. The identified binding sites were submitted along for docking. Docking parameters were determined using ATTRACTPep (accessed on August 10, 2022; http://www.attract.ph.tum.de/services/ATTRACT/peptide.html). The docking simulation process was completed on locally installed ATTRACT on the CHPC cluster for South Africa. Models generated with the lowest energy and ability to bind effectively to the receptor were selected. The Visual Molecular Dynamics (VMD) and Chimera software were used to visualize the docked structure.^63^ The LigPlot+ software was used to determine the two-dimensional interaction between the docked complex comprising the receptor and ligand.^78^

#### Molecular dynamics simulations

The docked complex comprising of MEV and TLR-2 was subjected to molecular dynamic simulation using AMBER 14 package.^79^ This process facilitates the determination of the stability and also their interactions.^80^ The input protein was described with the aid of FF14SB,^81^ and the LEAP module in AMBER 14 was used to generate the topologies of the vaccine structure through the addition of ions (protons and Cl^–^) to neutralize the systems.^79^ An orthorhombic box TIP3P water molecules was exclusively used as the solvent medium of the system, with the atoms within 8 Å from the box edges.^80^ To reduce energy configurations in the protein, initial energy minimization process was done with 10 000 steps (500 steepest descent with 9500 conjugate gradient) with subsequent full minimization at a lower level of 2000 steps. Gradual heating of the system was done from 0 to 300K in a canonical ensemble (NVT [constant number N, volume V, and temperature T]) of MD simulation for 2 ns using the Langevin thermostat with a collision frequency of 1.0 per second such that a fixed number of atoms and volume was maintained in the system. The density of water was controlled with 4 ns NVT simulation. The system was equilibrated at 300 K for 2 ns at a pressure of 1 bar. The production was run for 120 ns of NPT (constant number N, pressure P, and temperature T) using the GPU version provided in the Amber 18 package. Parameters such as RMSD and root mean square fluctuations (RMSF) were analysed using CPTRAJ and PPTRAJ modules in the AMBER 14 package.^82^ Visualization of the graphical representation of the complex was done using VMD v1.9.3 software. The end point free binding energy of the docked complex was calculated using the Molecular Mechanics/Generalized Born Surface Area (MM/GBSA) module in the AMBER 14 package with the formula, ΔG_bind_ = G_complex_ – (G_receptor_ + G_ligand_).

### Codon optimization and in silico cloning of the MEV

Codon optimization is vital for inducing the expression of a protein. This analysis used Java Codon Adaption (Jcat: http://www.jcat.de/)^83^ online web server. This process was performed to obtain the maximum expression using *Escherichia coli* codon system. The Codon adaption index (CAI) and CG content of the MEV are essential in evaluating gene expression. Ideal CAI and GC scores are between 0.8 and 1.0 and 30% to 70%, respectively, favouring translation and transcription processes.^67,84^ Cloning and visualization of the optimized sequence of the MEV were done using the Snapgene viewer tool v6.6 (http://www.snapgene.com/). These sequences were cloned into *E coli* (K-12 strain) plasmid PGL 4.10 vector. PstI (CTGCAG) and Hind III (AAGCTT) were used as restriction sites at both terminals of the MEV fragment.

## Results

### Protein sequence selection, retrieval, and filtering

The NCBI database was used exclusively to obtain the amino acid sequence of the RAP-1 protein in FASTA format. This protein is an integral part of *Babesia* species and is known to play key roles in the immunologic pathway of babesiosis. A total of 71 RAP-1 sequences across all bovine *Babesia* species were retrieved from the NCBI database and used to design a subunit MEV (see Supplementary Table 1). The MSA was done using CLUSTAL W online server to obtain conserved sequences. Conserved and unique sequences were identified from the analysis.

### Evaluation of antigenicity and topology (transmembrane status)

The unique conserved sequences were further tested for antigenicity with Vaxijen v2.0 set at a threshold of 0.4, with all other parameters set at default mode. A total of 38 sequences met the antigenic threshold requirement set earlier at 0.4. The transmembrane status of these antigenic sequences was analysed using the TMHMM v2.0 online web server, which detected 21 conserved sequences that exhibited outside transmembrane status (see Table 1).

**Table 1.** The conserved antigenic outside transmembrane sequence of *Babesia*.

S/No	Sequence	Antigenicity	Transmembrane status
1.	EGTTDVEYLVNKVLYMATMNYKTYLTVNSMNAKFFNRFSFTTKIFSRRIRQTLSDIIRWN	0.6395	Outside
2.	FLGVCFGALLLVARSGSAIRYTHRSGVMSAEVVGDVSKTLLAANEVVNAEMEAAQVNKD	0.5221	Outside
3.	FLSRYLFMTTIYYKTYTTIEKMKTSLQNNMKIARYLCSKRIRKALGNILKVN	0.5363	Outside
4.	IATEEETEPVEENKSVFGKVKEKLGNIRFNTGIFRKGEAKTRHSHLSEEDIMGSLSSADA	0.6541	Outside
5.	IATEEETEPVEENKSVFGKVKEKLGNIRFNTGIFRKGEAKTRHSHLSEEEIMGSLSSADA	0.6781	Outside
6.	LAFTTRLFGFGIQKALKRLVRSN	0.4376	Outside
7.	LPVDLGTHPEATIREIASGYGEYMMTQVPAMTSFAERFSKMATKTLLVTVSDYVHLPA	0.4960	Outside
8	LPVDLGTHPEATIREIASGYGEYMMTQVPAMTSFAERFSKMATKTLLVTVSDYVHLPT	0.4889	Outside
9.	LPVDLGTHPEATIREIASGYGEYMMTQVPAMTSFAERFSKMATKTLLVTVSDYVRLPA	0.5281	Outside
10	LPVVLGTHPEATIREIASGYGEYMMTLVPAMTSFAERFSKMATKTLLVTVSDYVHLPA	0.5275	Outside
11.	MQEEIGLINDDSIAEMCLGSKDEHHCASQIAAYVARCKEGNCLTIDAVGKPQNKAYEQLV	0.5344	Outside
12.	PESNDVENFASQYFYMTTLYYKTYLTVDFTAAKFFIKLAFTTRLFGFGIQKALKRLVRSN	0.6941	Outside
13.	SDHSEVEDLVNRYLYMATMYYKTYLILDTTKAHLINKIDFAHHIFGKSIKHMLEKIIRNH	0.4401	Outside
14.	SDNSEVEDLVNRYLYMATMYYKTYLILDTTKAHLINKIDFAHHIFGKSIKHMLEKIIRNH	0.4268	Outside
15.	SDNSEVEDLVNRYLYMATMYYKTYLILDTTKAHLINKIDFAHHVFGKSIKHMLEKIIRNH	0.4296	Outside
16.	SDNSEVEDLVNRYLYMATMYYKTYLILDTTKAHLINNIDFAHHIFGKSIKHMLEKIIRNH	0.5269	Outside
17.	VGDVSKTLLEANEVVNAEMEATQVNKD	0.4500	Outside
18.	VPEDFEERSIERITQLTSSYEDYMLTQIPTLSKFARRYADMVKKVLLGSLTSYVEAPW	0.4842	Outside
19.	VPEDFEERSIKRITQLTSSYEDYMLTQIPTLSKFARRYADMVKKVLVG	0.4210	Outside
20.	VPEHFEETSNERITQLTSSYEDYMLTVIPSHSKFARRYADMVKKVLLG	0.7567	Outside
21.	YLVNKVLYMATMNYKTYLTVNSMNAKFFNRFSFTTKIFSRRIRQTLSDIIRWN	0.6275	Outside

### Prediction of T- and B-cell epitope

#### Cytotoxic T-lymphocyte (CTL/CD8^+^) epitopes and MHC-1 binding alleles

The conserved and exomembrane 22 sequences were used to generate nonamers of CD8^+^ epitopes using the NetCTL v1.2 online tool. A total of 978 highly immunogenic nonamers of CD8^+^, which are receptor specific were obtained. The nonamers obtained were further subjected to the IEDB MHC-1 prediction online server for analysis. The SSM-based analysis method was done with the IC_50_ score parameter set at < 250 to obtain binding alleles accurately. The results from the analysis showed a total of 346 CD8^+^ epitopes that interacted with the MHC alleles set earlier. Antigenicity testing for the selected CD8^+^ epitopes was done using Vaxijen v2.0 at a higher threshold of 0.5, with all other parameters in default mode. A total of 113 CD8^+^ epitopes were antigenic, with the threshold value ranging from 2.0506 to 0.5510. The sequence FTTKIFSRR had the highest antigenic value score but was screened out as allergenic during the allergenicity test. The IEDB tool was used for immunogenicity analysis, which detected 15 sequences as being conserved. Further allergenicity testing of the conserved sequences using AllerTop v2.0 revealed 11 sequences to be non-allergens. These sequences include KIDFAHHVF, TYLTVDFTA, SQIAAYVAR, FTAAKFFIK, FIKLAFTTR, LTSYVEAPW, AAKFFIKLA, FMTTIYYKT, YMMTLVPAM, TAAKFFIKL, and SQYFYMTTL. These are the final predicted CD8^+^ epitopes. The overview of results for final CD8^+^ epitopes with their properties in terms of HLA alleles, IC_50_ value, antigenicity, and allergenicity is shown in Table 2.

**Table 2.** Final analysed CD8^+^ T-cell epitopes, which were 100% overlapped with CD4^+^ T-cell epitopes and interacted with different MHC-I alleles.

Epitopes	HLA alleles	IC_50_	Antigenicity	Allergenicity
KIDFAHHVF	HLA-A*32:01	49.53	1.1037	Non-allergen
TYLTVDFTA	HLA-A*23:01	202.96	0.5356	Non-allergen
SQIAAYVAR	HLA-A*31:01	53.76	0.6268	Non-allergen
FTAAKFFIK	HLA-A*68:01	13.07	0.9852	Non-allergen
	HLA-A*11:01	26.07		
	HLA-A*31:01	165.37		
	HLA-A*03:01	168.18		
FIKLAFTTR	HLA-A*68:01	72.86	1.5344	Non-allergen
	HLA-A*31:01	90.67		
	HLA-A*33:01	115.35		
LTSYVEAPW	HLA-A*68:01	06.04	1.1744	Non-allergen
	HLA-B*57:01	48.73		
AAKFFIKLA	HLA-A*30:01	108.88	0.9670	Non-allergen
IAAYVARCK	HLA-A*68:01	76.29	0.8741	Non-allergen
FMTTIYYKT	HLA-A*02:03	125.01	1.6065	Non-allergen
	HLA-A*02:01	164.10		
CASQIAAYV	HLA-A*68:02	64.50	0.6534	Non-allergen
YMMTLVPAM	HLA-A*02:01	7.58	0.7904	Non-allergen
	HLA-B*15:01	16.48		
	HLA-B*35:01	20.87		
	HLA-A*02:03	21.47		
	HLA-A*02:06	29.89		
	HLA-B*08:01	147.75		
TAAKFFIKL	HLA-A*68:02	122.06	0.7402	Non-allergen
SQYFYMTTL	HLA-B*15:01	181.97	1.0044	Non-allergen
	HLA-A*32:01	89.11		
	HLA-A*32:01	128.80		

### Helper T-lymphocytes (HTL/CD4^+^) epitopes and MHC-II binding alleles

The 22 conserved sequences that fulfil the requirement for transmembrane analysis were further subjected to MCH-II binding analysis using IEDB online server to predict CD4^+^ T-cell epitopes and HLA alleles, respectively. This analysis was set at IC_50_ value < 250. Further antigenicity tests were conducted on the epitopes using Vaxijen v2.0 set at 0.5 thresholds. A total of 1091 epitopes successfully passed the required values set for the IEDB and antigenicity test set earlier. These CD4^+^ T-cell epitopes were analysed for the conservancy, where 52 CD4^+^ T-cell epitopes were conserved. IFNepitope and IL-4pred immunoinformatics tools were used to identify conserved CD4^+^ T-cell epitopes that can finally activate immunologic response by producing cascade signalling cytokines. A total of 27 CD4^+^ T-cell epitopes were identified as IFN-gamma and IL-4 inducers. The allergenicity test of the epitopes using AllerTop v2.0 identified 17 CD4^+^ T-cell epitopes as non-allergenic. These were finally considered the CD4^+^ T-cell epitopes for designing the sub-unit MEV (see Table 3).

**Table 3.** Final analysed CD4^+^ T-cell epitopes, 100% overlapped with CD8^+^ T-cell epitopes and interacted with different MHC-II alleles.

Epitope	HLA alleles	IC_50_	Antigenicity	Allergenicity
ASGYGEYMMTLVPAM	HLA-DRB1*01:01	65.00	0.8432	Non-allergens
	HLA-DRB1*01:01	44.00		
	HLA-DRB1*04:01	218.00		
DEHHCASQIAAYVAR	HLA-DRB1*01:01	231.00	0.6600	Non-allergens
DFAHHVFGKSIKHML	HLA-DRB1*07:01	84.00	0.8075	Non-allergens
	HLA-DRB1*11:01	108.00		
DVENFASQYFYMTTL	HLA-DRB1*01:01	243.00	0.6411	Non-allergens
EHHCASQIAAYVARC	HLA-DRB1*01:01	238.00	0.6056	Non-allergens
ENFASQYFYMTTLYY	HLA-DRB5*01:01	207.00	0.8525	Non-allergens
	HLA-DRB1*01:01	63.00		
	HLA-DRB1*04:01	139.00		
	HLA-DRB1*11:01	173.0		
	HLA-DRB1*04:05	130.00		
FMTTIYYKTYTTIEK	HLA-DRB1*15:01	183.00	0.6720	Non-allergens
	HLA-DRB1*04:05	158.00		
FTAAKFFIKLAFTTR	HLA-DRB1*01:01	41.00	1.1569	Non-allergens
	HLA-DRB1*11:01	202.00		
GYGEYMMTLVPAMTS	HLA-DRB1*04:05	190.00	0.5175	Non-allergens
	HLA-DRB1*11:01	231.00		
IDFAHHVFGKSIKHM	HLA-DRB1*07:01	236.00	0.7131	Non-allergens
KVLLGSLTSYVEAPW	HLA-DRB1*04:05	106.00	1.1597	Non-allergens
	HLA-DRB1*15:01	209.00		
LTVDFTAAKFFIKLA	HLA-DRB5*01:01	121.00	0.6201	Non-allergens
	HLA-DRB1*07:01	66.00		
SQIAAYVARCKEGNC	HLA-DRB5*01:01	164.00	1.0453	Non-allergens
TVDFTAAKFFIKLAF	HLA-DRB5*01:01	126.00	0.7477	Non-allergens
	HLA-DRB1*07:01	205.00		
TYLTVDFTAAKFFIK	HLA-DRB1*07:01	71.00	0.6222	Non-allergens
	HLA-DRB5*01:01	126.00		
VDFTAAKFFIKLAFT	HLA-DRB1*07:01	210.00	0.7449	Non-allergens
	HLA-DRB5*01:01	129.00		
YLTVDFTAAKFFIKL	HLA-DRB1*07:01	65.00	0.7071	Non-allergens
	HLA-DRB5*01:01	122.00		

### Population coverage analysis

**Figure 1.**
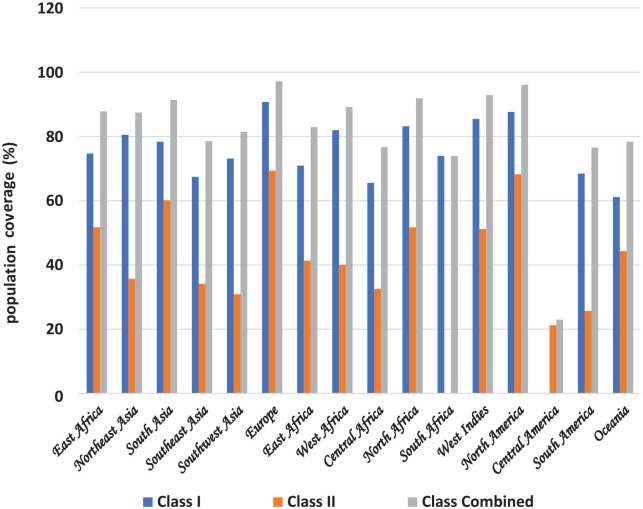
The population coverage analysis of individual and combined epitopes across the globe.

The global population coverage of T-cell epitopes is presented graphically in Figure 1 (see Supplementary Table 2). The global Class combined HLA coverage value is 81.58%. The Class combined value is highest in Europe (97.16%), followed by North America (96.07%), West Indies (92.89%), North Africa (91.88%), South Asia (91.37%), West Africa (89.17%), East Asia (87.79%), Northeast Asia (87.48%), East Africa (82.95%), Southwest Asia (81.43%), Southeast Asia (78.55%), Oceania (78.37%), Central Africa (76.75%), South America (76.55%), South Africa (76.75%), and Central America (22.94%).

### B-cell epitopes

The ABCpred online server was used to identify linear B-cell epitopes set at 0.51 threshold with a window length of 16. The 22 conserved sequences that passed the requirement of the transmembrane status were further used for the analysis and uploaded to the online server. A total of 80 B-cell epitopes were obtained from the analysis and further subjected to the following tests. These tests include antigenicity, allergenicity, and conservancy. Three B-cell epitopes passed the various tests with results above the minimum set criteria. They were included in the final predicted epitopes to be used in designing the sub-unit MEV (see Table 4).

**Table 4.** Final predicted B-cell epitopes with their properties.

Epitopes	ABC predicted score	Position	Antigenicity	Conservancy	Allergenicity
TLYYKTYLTVDFTAAK	0.74	624	0.6248	100%	Non-allergen
ASQIAAYVARCKEGNC	0.89	573	0.9955	100%	Non-allergen
TVDFTAAKFFIKLAFT	0.75	632	0.5947	100%	Non-allergen

### Molecular docking of T-cell epitopes to MHC

Docking of T-cell epitopes (CD8^+^/CD4^+^) to the active site of their most consistent HLA alleles was done. These involved the determination of their flexibility through the use of ATTRACTpep, and further docking simulation facilitated using CHPC South Africa. A total of 50 frames were generated for each epitope after successful binding to corresponding HLA alleles. However, only the complexes with the least binding energy were selected. The binding energy level for the CD8^+^ epitopes ranged from −112.959 to −139.399 kcal/mol, while that of CD4^+^ is within −113.373 to −164.890 kcal/mol as shown in Table 5.

**Table 5.** The binding energy levels between the interaction of T-cell epitopes with their HLA alleles.

CD8^+^ T-cell epitopes	Energy (kcal/mol)
KIDFAHHVF	−132.357
TYLTVDFTA	−137.822
SQIAAYVAR	−109.383
FTAAKFFIK	−139.399
FIKLAFTTR	−154.295
LTSYVEAPW	−134.989
AAKFFIKLA	−112.959
IAAYVARCK	−116.151
FMTTIYYKT	−119.057
CASQIAAYV	−130.608
YMMTLVPAM	−116.143
TAAKFFIKL	−138.526
SQYFYMTTL	−132.365
CD4^+^ T-cell epitopes	
ASGYGEYMMTLVPAM	−113.373
DEHHCASQIAAYVAR	−116.307
DFAHHVFGKSIKHML	−129.268
DVENFASQYFYMTTL	−124.265
EHHCASQIAAYVARC	−150.199
ENFASQYFYMTTLYY	−127.263
FMTTIYYKTYTTIEK	−159.465
FTAAKFFIKLAFTTR	−164.890
GYGEYMMTLVPAMTS	−141.670
IDFAHHVFGKSIKHM	−145.817
KVLLGSLTSYVEAPW	−126.195
LTVDFTAAKFFIKLA	−142.561
SQIAAYVARCKEGNC	−163.929
TVDFTAAKFFIKLAF	−147.391
TYLTVDFTAAKFFIK	−131.588
VDFTAAKFFIKLAFT	−134.857
YLTVDFTAAKFFIKL	−141.243

### Design of multi-epitope sub-unit vaccines

**Figure 2.**
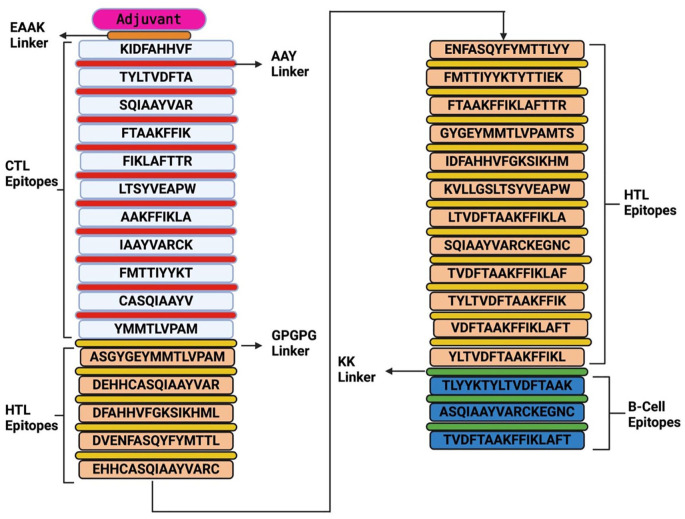
Designed multi-epitope vaccine comprising of epitopes, linkers, and adjuvant. CTL indicates cytotoxic T-lymphocyte; HTL, helper T-lymphocyte.

The final predicted epitopes chosen based on their antigenic, non-allergenic, immunogenic, conserved, and active cytokine inducers were used to design the sub-unit MEV construct. The final predicted epitopes include 11 CTL epitopes, 17 HTL epitopes, and 3 B-cell epitopes. The AAY, KK, and GPGPG linkers were used to link the CTL, HTL, and B-cell epitopes, respectively (see Figure 2). Beta-defensin-3 sequence (Q5U7J2) was incorporated as an adjuvant to potentiate the immunogenicity of the vaccine. An adjuvant was included and attached at the N-terminal of the MEV in connection with the EAAK linker. The constructed MEV vaccine comprises amino acids. The final sub-unit vaccine was further subjected to various software testings to determine its antigenicity, allergenicity, and physicochemical properties.

### Evaluation of multi-epitope sub-unit vaccine

#### Antigenicity, allergenicity, solubility prediction, physicochemical properties, and secondary structure

**Figure 3.**
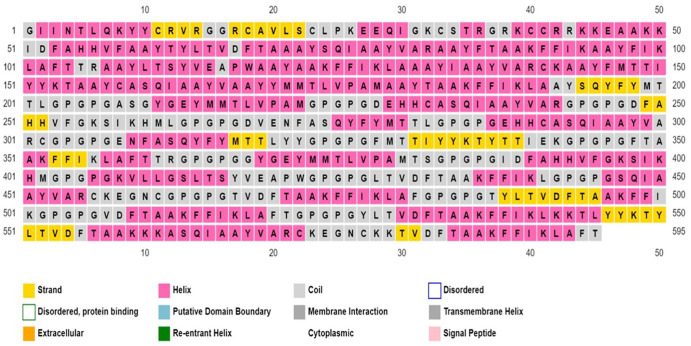
Secondary structure analysis of the multi-epitope vaccine construct.

Multi-epitope vaccine construct was subjected to numerous analyses using online software to validate its suitability as an effective vaccine. Results from the analysis showed that the vaccine was antigenic (Vaxijen score:0.6), non-allergen, and soluble (solubility index: 0.730) making it a possible vaccine candidate. Evaluation of the physicochemical properties of the MEV (see Table 6) using the Protparam server estimated that the MEV is composed of 595 amino acids, a molecular weight of 64 152 Daltons, theoretical isoelectric point (pI) of 9.52 and an instability index (II) of 13.89. The instability index value indicates the stability of the protein. The aliphatic index of 65.82 suggests the MEV’s ability to withstand high-temperature levels, and a GRAVY index value of 0.122 indicates the vaccine is thermostable and hydrophobic, respectively. The N-terminal of the sequence is G (Gly), with the half-life value in hours estimated to be 30 in mammalian reticulocytes (*in vitro*), > 20 in yeasts (*in vivo*), and > 10 in *E coli* (*in vivo*). A higher number of positive residues than negative residues was seen during the analysis. The overall result of the physicochemical analysis validates the immunogenic status of the MEV designed. Analysis of the secondary structure of the MEV developed (see Figure 3) showed the vaccine is composed of 28.24% alpha helix, 29.92% extended strand, 3.70% beta-turn, and 38.5% random coil.

**Table 6.** Physicochemical properties of the multi-epitope subunit vaccine computed by the Protparam web server.

Physicochemical properties for MEV	Values
Number of amino acids	595
Molecular weight	6415.66
Theoretical pI	9.52
Total number of negatively charged residues (Asp + Glu)	27
Total number of positively charged residues (Arg + Lys)	63
Total number of atoms	9000
Ext. coefficient	81,905
The instability index (II)	13.89
Aliphatic index	65.82
Grand average of hydropathicity (GRAVY)	0.122

Abbreviations: MEV, multi-epitope vaccine; pI, isoelectric point.

### Prediction, refinement, and validation of tertiary structure

**Figure 4.**
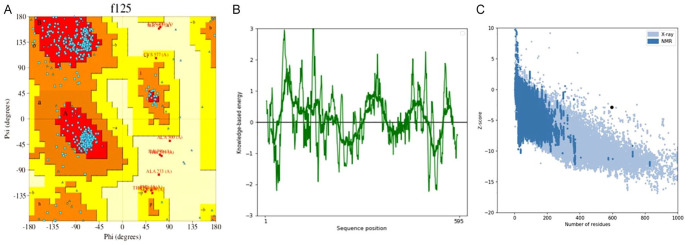
(A) Ramachandran plot of the refined multi-epitope vaccine showing 87.1% of residues in the favoured region. (B) Energy plot and (C) PROSA web plot showing an overall Z-score of −2.88.

The tertiary structure analysis of the MEV was generated using the Galaxy web server. The structure refinement was done using the Galaxy Refine web server to improve its consistency by enhancing loop refinement and energy functions. The galaxy refine server-generated five models based on structural factors such as GDTA-HA, RMSD, and MolProbity. Model 4 was selected as the best due to its favourable values obtained from the analysis, such as GDT-HA (0.9878), RMSD (0.274), MolProbity (2.199), Clash score (15.9), and poor rotamers (0.7) and Rama favoured (81.7%). Further validation and confirmation of the structure was done using the PROCHECK server and ProSA-web server. PROCHECK server generated a Ramachandran plot (see Figure 4A), which showed that 87.1% of the amino acids were found in the most favoured regions, 10.8% additional allowed regions, and 0.8% generously allowed regions. The energy plot values in relation to the residues are within the normal range (see Figure 4B). Validation of the selected model using ProSA-web estimated a Z-score of −2.88 (see Figure 4C)

### Discontinuous (conformational) B-cell epitopes

**Figure 5.**
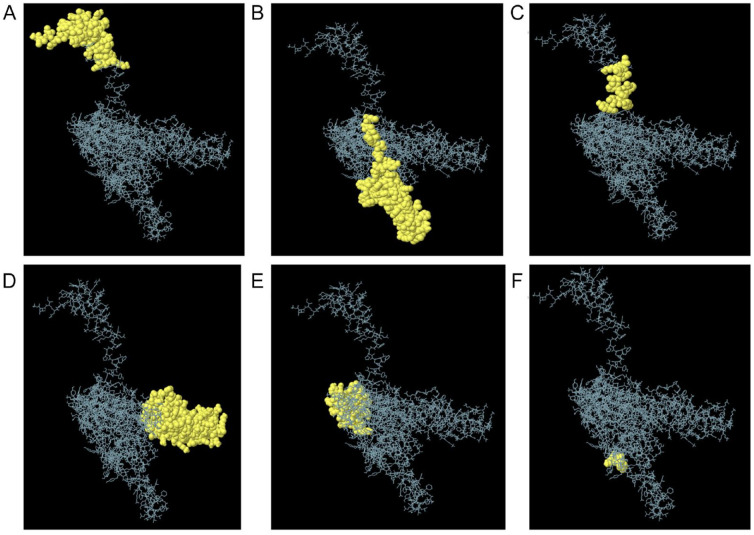
Conformational B-cell epitopes of the multi-epitope vaccine. The yellow colour spheres represent the three-dimensional (3D) appearance of the discontinuous B-cell epitopes, while the skeleton is described with a grey appearance. (A) Forty-seven residues showing a score of 0.9, (B) 82 residues showing a score of 0.8, (C) 14 residues showing a score of 0.758, (D) 110 residues showing a score of 0.674, (E) 56 residues showing a score of 0.548, and (F) 3 residues showing a score of 0.542.

A total of 312 residues were predicted using the Ellipro tool into six epitopes (see Table 7) and were considered discontinuous epitopes. The predicted conformational B-cell epitope score ranged from 0.542 to 0.8. The conformational B-cell epitope was predicted and shown graphically (see Figure 5).

**Table 7.** Predicted residues showing conformational B-cell epitopes of the MEV.

No.	Residues	No. of residues	Score
1.	A:G1, A:I2, A:I3, A:N4, A:T5, A:L6, A:Q7, A:K8, A:Y9, A:Y10, A:C11, A:R12, A:V13, A:R14, A:G15, A:G16, A:R17, A:C18, A:A19, A:V20, A:L21, A:S22, A:C23, A:L24, A:P25, A:K26, A:E27, A:E28, A:Q29, A:I30, A:G31, A:K32, A:C33, A:S34, A:T35, A:R36, A:G37, A:R38, A:K39, A:C40, A:C41, A:R43, A:K44, A:E46, A:A47, A:A48, A:K49	47	0.924
2.	A:L64, A:T65, A:V66, A:D67, A:F68, A:T69, A:A70, A:A71, A:A72, A:Y73, A:S74, A:Q75, A:I76, A:A77, A:A78, A:Y79, A:V80, A:A81, A:R82, A:A83, A:A84, A:Y85, A:F86, A:T87, A:A88, A:A89, A:K90, A:F91, A:F92, A:I93, A:K94, A:A95, A:A96, A:Y97, A:F98, A:I99, A:K100, A:L101, A:A102, A:F103, A:T104, A:T105, A:R106, A:A107, A:A108, A:Y109, A:L110, A:T111, A:S112, A:Y113, A:V114, A:E115, A:A116, A:P117, A:W118, A:A119, A:A120, A:Y121, A:A122, A:A123, A:K124, A:F125, A:F126, A:I127, A:K128, A:L129, A:A130, A:A131, A:A132, A:Y133, A:I134, A:A135, A:A136, A:Y137, A:V138, A:A139, A:R140, A:C141, A:K142, A:A143, A:A144, A:F146	82	0.8
3.	A:K50, A:I51, A:D52, A:F53, A:A54, A:H55, A:H56, A:V57, A:F58, A:A59, A:A60, A:Y61, A:T62, A:Y63	14	0.758
4.	A:G484, A:G486, A:T487, A:Y488, A:L489, A:T490, A:V491, A:D492, A:F493, A:T494, A:A495, A:A496, A:K497, A:F498, A:F499, A:I500, A:G502, A:P503, A:G504, A:P505, A:G506, A:V507, A:D508, A:F509, A:T510, A:A511, A:A512, A:K513, A:F514, A:F515, A:I516, A:K517, A: L518, A:A519, A:F520, A:T521, A:G522, A:P523, A:G524, A:P525, A:G526, A:Y527, A:L528, A:T529, A:V530, A:D531, A:F532, A:T533, A:A534, A:A535, A:K536, A:F537, A:F538, A:I539, A:K540, A:L541, A:K542, A:K543, A:T544, A:L545, A:Y546, A:Y547, A:K548, A:T549, A:Y550, A:L551, A:T552, A:V553, A:D554, A:F555, A:T556, A:A557, A:A558, A:K559, A:K560, A:K561, A:A562, A:S563, A:Q564, A:I565, A:A566, A:A567, A:Y568, A:V569, A:A570, A:R571, A:C572, A:K573, A:E574, A:G575, A:N576, A:C577, A:K578, A:K579, A:T580, A:V581, A:D582, A:F583, A:T584, A:A585, A:A586, A:K587, A:F588, A:F589, A:I590, A:K591, A:L592, A:A593, A:F594, A:T595	110	0.674
5.	A:F310, A:A311, A:S312, A:Q313, A:Y314, A:F315, A:Y316, A:M317, A:T318, A:T319, A:L320, A:Y321, A:Y322, A:G323, A:P324, A:G325, A:F328, A:T330, A:T331, A:K342, A:G343, A:P344, A:G345, A:P346, A:T349, A:R362, A:G363, A:V377, A:P378, A:A379, A:M380, A:T381, A:S382, A:G383, A:P384, A:G385, A:P386, A:G387, A:I388, A:D389, A:F390, A:A391, A:H392, A:H393, A:V394, A:F395, A:G396, A:K397, A:S398, A:I399, A:K400, A:H401, A:M402, A:G403, A:P404, A:G405	56	0.548
6.	A:Y145, A:T149, A:K153	3	0.542

Abbreviation: MEV, multi-epitope vaccine.

### Protein disulfide bond engineering of the MEV

**Figure 6.**
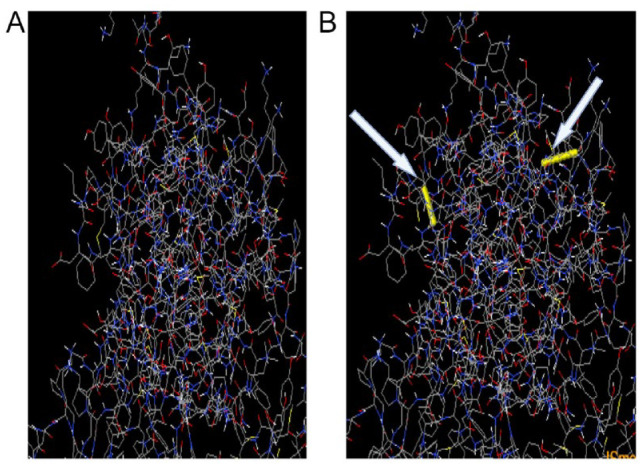
Disulfide bond formation of the multi-epitope vaccine showing (A) original and (B) mutant. Yellow colour rod-like structures represent the disulfide bond formed denoted by white arrows.

A total of 30 pairs of amino acid residues were identified as capable of forming disulfide bonds based on the criteria for the disulfide engineering set earlier using the Disulfide by Design v2.12 online server. Disulfide bonds were produced using the 3D structure of the multi-epitope vaccine after refinement. However, only two pairs whose energy bond level score was less than 2.2 kcal/mol was used for the disulfide bond formation, including Cyst11–Cyst40 and Ala250–Gly255, for which the χ3 angles are +104.02 and +95.27. The MEV was converted to the mutant form using the create-mutant button on the online server (see Figure 6).

### Immune simulation

**Figure 7.**
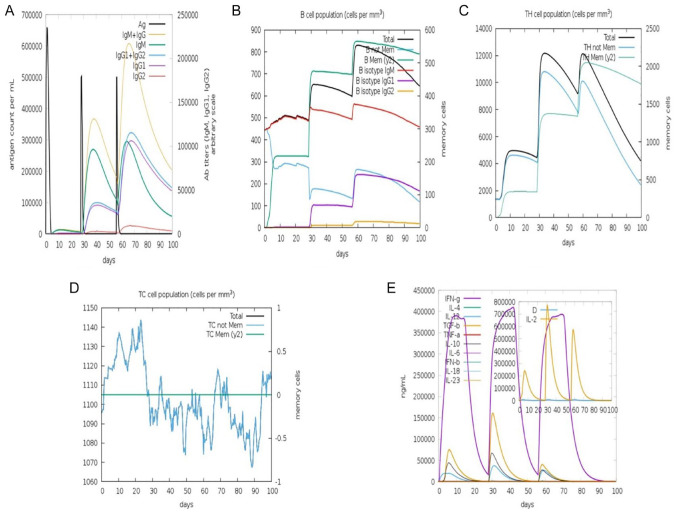
Immunogenic profile of the MEV after immune simulation using C-IMMSIM online server. (A) Immunoglobulin response of the MEV is injected. Black vertical lines represent the antigen, while subtypes appear multicoloured. (B) The B-lymphocytes population is categorized into Igl, Ig2, and IgM. (C) Progressive phase of T-helper cells. (D) Cytotoxic T-cell population after exposure to the antigen. (E) The concentration of interleukins and cytokines after exposure to the antigen. Ig indicates immunoglobulin; MEV, multi-epitope vaccine; TC, T-cytotoxic; TH, T-helper.

The success of a vaccine construct largely depends on the immunogenic vaccine profile, which was measured using C-IMMSim online web server. This simulation mimics the immune system response in a patient when the sub-unit vaccine is administered. The results shown in Figure 7A to E depict that the sub-unit vaccine can induce a cellular and humoral response. The immunogenic profile showing the cellular and humoral response of the vaccine constructed is shown in Figure 7. There was an insignificant immune response after the first exposure to the vaccine candidate. However, a significant increase in the antibody titre was observed in the next two successive vaccinations (2 and 3). An increase in immunoglobulin activity was seen with a decrease in antigenic concentration after secondary and tertiary immune response (see Figure 7A). This shows an inverse relationship between immunoglobulin activity and antigen concentration. The population of B-cell memory (see Figure 7B) showed a rapid increase in the cells with a corresponding decline in non-memory cells. The B-cell response increased after each exposure to the MEV indicating a high secondary immune response mechanism. An increase in CD4^+^ T-cell population was observed after each exposure to the vaccine (see Figure 7 C). However, the total CD4^+^ T-cell population remained constant after the administration of the third dose of the vaccine. In Figure 6D, a constant response of non-memory cells was observed with increased CD8^+^ T cell memory cells after an initial decrease. The vaccine was able to increase the production and concentration of important cytokines (IL-10, IL-12, interferon gamma [interferon-γ], and transforming growth factor-beta [TGF-β]) after administration of each vaccine dose (see Figure 7E). An initial increase in concentration was observed after administration of the first two vaccines; however, a decrease in the concentration was observed after the third dose was given.

### Molecular docking of MEV construct to the receptor (TLR-2) and molecular dynamic simulations

Molecular docking was done to analyse the binding interaction with the receptor (TLR-2) PDB ID:5D3I to the vaccine construct. Fifty docked complexes were produced and visualized using VMD and Chimera software to understand the interaction and binding affinity clearly. The least binding energy of −190.269 was selected as the top docked complex among all the different complex conformations.

**Figure 8.**
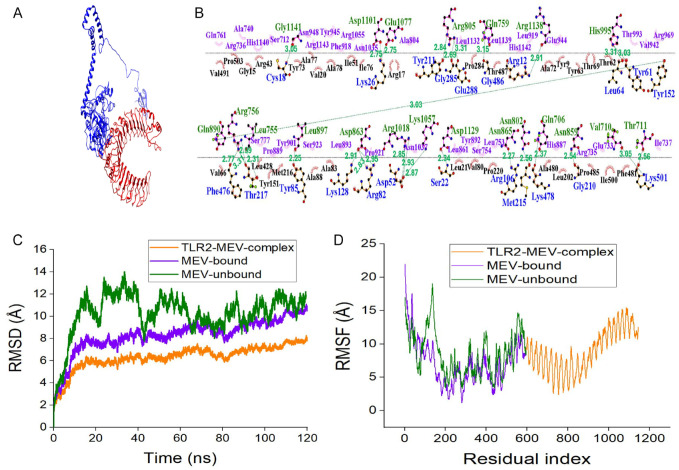
(A) The docked TLR-2–MEV complex. TLR-2 is shown in red, while the MEV is represented in blue. (B) TLR-2–MEV docked interaction as analysed by LigPlot+. The green colour represents the receptor residue interacting with MEV, while the blue denotes the MEV. Residues in purple and black represent the hydrophobic interaction. (C) The molecular dynamic simulation of the MEV–TLR-2 docked complex (golden), MEV bound with TLR-2 (purple) and MEV-unbound (green). The RMSD plot highlighting the stability of the structure through analysis of the backbone. The MEV-bound to TLR-2 showed more stability when compared to the unbound MEV. (D) The RMSF plot showing interactions of the sidechain atoms. MEV indicates multi-epitope vaccine; RMSD, root mean square deviation; RMSF, root mean square fluctuation; TLR-2, Toll-like receptor-2.

The docked TLR-2–MEV complex from molecular docking (see Figure 8A) was submitted for molecular dynamic simulation. The result of the two-dimensional interaction between the receptor and the ligand showing the residues is graphically presented in Figure 8B. The RMSD values of the TLR-2–MEV complex (golden) and MEV-bound (purple) is lower than the MEV-unbound (green; see Figure 8C). The MEV-bound refers to the MEV after being bound to the TLR-2, while MEV-unbound refers to the free MEV that has not interacted with TLR-2. The atom back bone fluctuations of the bound-MEV ranges from 2.5 to 11.0 Å, while the atoms of the MEV-unbound from 0.0 to 12.2 Å. The average RMSD observed for the complex is 6.2 Å, which is lower than the one observed in some studies where values of 1.08 ± 0.1 and 0.95 ± 0.14 nm were reported, respectively.^70,71^ The lower RMSD observed for the MEV-bound than that of the MEV-unbound indicates enhanced stability on binding with TLR-2, which is advantageous to vaccine design. It was also observed that the TLR-2–MEV complex is more stable between 20 and 60 ns with an average of 5.9 Å. A lower fluctuation level was observed for TLR-2–MEV complex and MEV bound compared to MEV-unbound on the RMSF plot (see Figure 8D). Estimation of the binding affinity (–139.98 kcal/mol) of the MEV to TLR-2 was done using the MM/GBSA (see Supplementary Table 3). The values for the endpoint energy between their interactions is favourable due to the negative van der Waals energy value and a significant contribution can be observed from electrostatic gas phase and solvent free energy.

### In silico cloning and codon optimisation

**Figure 9.**
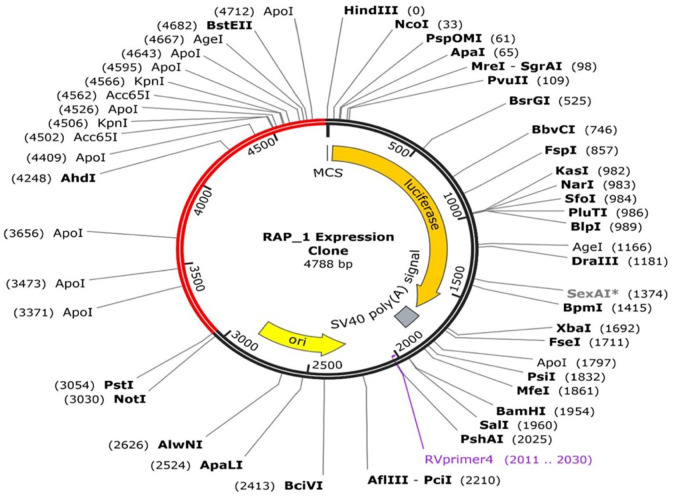
*In silico* cloning of the multi-epitope vaccine into a vector (PGL 4.10). RAP-1 indicates rhoptry-associated protein 1.

Reverse translation of the protein sequence into nucleotide sequence was done with Jcat, an online server tool. A 4788 bp in length DNA sequence was constructed. The software predicted the CAI value and the GC content value of 0.9% and 52.8%, respectively, which showed a good expression of the MEV produced in the *E coli* K12 strain as it favours translation and transcription. Snapgene free trial version was used for *in silico* cloning of the improved sequence where pstI and Hind III were employed as restriction sites at the terminals, which was finally cloned into PGL 4.10 vector plasmid, as presented in Figure 9.

## Discussion

Bovine babesiosis is one of the most cosmopolitan tick-borne infections associated with a substantial impact on human and animal health.^10,85^ A global population of 500 million cattle is at risk of infection, especially in regions where the parasite and the tick vector exist. Despite the various strategies used to control babesiosis, an increase in the prevalence of the infection is still observed owing largely to ineffective preventive measures such as the limitation of the live vaccines and the development of acaricidal resistance.^18^ The most reliable method for the control of any pathogen is vaccination. However, the development processes of vaccines are protracted and costly. These lead to the application of bioinformatics for developing vaccines that save time and are cost-effective in terms of resources. The introduction of *in silico* analysis has facilitated the identification of potential vaccine candidates by analysing the pathogen’s genomic information and selecting novel antigens utilized for vaccine development. The growth in the field of bioinformatics, especially the reverse vaccinology approach, has led to the development of various sub-unit vaccines against various pathogenic diseases such as theileriosis,^86^ malaria,^87^ coccidiosis, and tuberculosis with further validation from the laboratory in recent times. This study was designed to develop an MEV from RAP-1, which can induce cellular and humoral responses against babesiosis without any pathology to the host. An important recipe in designing the MEV is identifying a biomarker or protein that plays a vital role in immunological response. The RAP-1 protein plays an essential role in erythrocytic invasion by the parasite and is considered a key target in designing vaccines.^88^ The immunologic response process of an individual mainly involves T lymphocytic cells and B lymphocytic cells once a foreign body (antigen) is identified by the antigen-presenting cell (APC). The APC further presents the antigen via MHC class II found on the surface of the helper T cells. These helper cells contain an important molecule on their surface called CD4. Finally, it activates B-cells, CD8^+^, and macrophages leading to the production of antibodies and the destruction of target antigens. Epitope prediction and selection involved the use of IEDB and ABCpred server. These online servers are accurate and reliable and have been proven to develop long-lasting vaccines with their application in several studies.^89^ The IEDB server is used in T-cell and B-cell epitope prediction, while the B-cells are selected using the ABCpred server. A further combination of various analyses, which involves antigenicity, allergenicity, and conservancy with their accuracy ranging from 65% to 88% was used in the study but further validation is required. The T-cells are categorized into cytotoxic (CTL) or helper T-lymphocytes (HTL). To ensure that CTL and B cells function effectively, HTL secretes cytokines as a modulator.^90^ The CTL is vital in responding against pathogens and abnormal cells by destroying infected cells.^91^ For a vaccine to be effective, it should be able to stimulate both cellular (CTL and HTL) and humoral (B-cell) responses against an infectious agent. Therefore, this study explored sequences conserved from the RAP-1 antigen to generate 11 CD8^+^, 17 CD4^+^, and 3 B-cell epitopes. These epitopes were confirmed to be antigenic, immunogenic, soluble, and highly conserved. Screening of CD4^+^ cells was done to evaluate their ability to produce key cytokines. Interferon-γ and IL-4 are important cytokines that play a key role in activating danger signalling cells via a stimulatory response. The B-cell epitopes could either be linear, which are contiguous in structure and mostly found in the primary structure of a protein, or discontinuous B-cell epitopes resulting from a close association by protein folding. An important function of the B-cell epitopes is the production of immunoglobulins, which makes these cells essential in designing MEVs.^92-94^ A promising characteristic is the high population coverage of the class combined epitopes across all regions, which indicates some degree of effectiveness and global coverage. An MEV was designed comprising of all epitopes in combination with an adjuvant using various linkers. The effectiveness of a vaccine largely depends on the adjuvant incorporated into the sub-unit vaccines. These adjuvants, in general, facilitate the host immune response through increase in antibody production.^95^ Beta-defensin-3 was added to the construct as an adjuvant using EAAK linker at the N-terminal. The choice of Beta-defensin-3 as an adjuvant was influenced based on its high protein folding and flexibility, resulting in a better vaccine candidate.^96^ Also, several studies have reported the adjuvant as an important immunomodulator with antiviral and antimicrobial effects.^97^ Beta-defensin-3 plays a key role in the resolution of inflammation, and its expression stimulates responses IL-1, interferon-γ, tumour necrotizing factor (TNF), and TLR ligands following injury or infection.^98-100^ They also play a key role in linking innate and adaptive immunity via the chemoreceptor 6 (CCR6) pathway.^101^ Beta-defensin-3 is an antimicrobial peptide that destroys pathogens that breach the membrane barrier.^102^ It is a broad antimicrobial that targets a large number of microorganisms.^103^ An increase in their expression has been directly associated with the stimulation of IL-1, TNF-α, LPS,^104^ IL-22,^105^ and flagellin.^106^ A combination of these cytokines further elicits the stimulation of IFN-γ^107^ and TLR-2.^108^ Beta-defensin-3 plays a key role in the induction of acquired immune response by recruiting immature dendritic cells, monocytes, and T-cells.^109^ They have also been implicated in the chemotaxis of dendritic cells via their interaction with chemokine receptor 6 (CCR6) and inducing the expression of costimulatory molecules on monocytes through their interaction with TLR-2.^110^ This natural protein has shown promise when used in non-rejectable peptide-based therapeutics due to its interaction with components of lipid membrane.^97,99,111^ This adjuvant has been used successfully to design vaccines *in silico* against several pathogens such as *Acinetobacter baumannii*,^103^ SARS-CoV-2,^112^ respiratory syncytial virus,^113^ Nipah virus,^114^ avian leukosis virus^115^ and Esptein-Barr virus.^116^

The CTL, HTL, and B-cell epitopes were linked using AAY, GPGPG, and KK linkers. The subunit MEV has been found to be antigenic, immunogenic, and non-allergenic after screening with IEDB, Vaxijen, and AllerTop online servers. These requirements are essential for the vaccine construct to be regarded as a potential vaccine candidate. The MEV has a molecular weight of 64 152 kDa with an instability index value of 13.89 and aliphatic index of 65.82 comprising of 595 amino acids. Molecular weight values < 110 kDa are beneficial in vaccine development owing to its rapid purification.^117,118^ Proteins with instability index value < 40 are regarded as thermostable and meet the standard requirement in vaccine development.^59,119^ Several studies reported varying figures on the length of amino acids in designing MEV against parasitic infections such as leishmania (528), malaria (541), and coccidiosis (731).^89,120,121^ Variations in the length of amino acids exist. However, this study’s length of amino acids is within the specified range documented in earlier research. Evaluation of the physicochemical properties of MEV showed the vaccine to be thermostable and hydrophobic with a GRAVY value of 0.122. The positive GRAVY value indicates the hydrophobic nature of the vaccine construct. Several advantages have been associated with positive GRAVY value. These include strong interaction with amino acid residues, smooth purification, enhanced protein stability, adding structural integrity vital for maintaining functional confirmation during administration and facilitating the presentation of cells essential for immune response.^122,123^ However, a significant limitation of hydrophobic vaccines is poor viable interaction with water, indicating its non-solubility. A strong binding affinity of the vaccine construct and TLR is necessary to facilitate the transmission of the vaccine construct into the body. Surfactants can be added to the vaccine during its development to enhance solubility. Surfactants are amphiphilic molecules comprising a lipophilic part linked to a hydrophilic part.^124^ Surfactants have physicochemical properties of solubilization or emulsification. These substances can be added to vaccines as adjuvants, solubilizers, or surfactants. The secondary structure of the proteins plays a crucial role in protein folding and provides information regarding protein function.^125^ The high percentage of random coils in the vaccine construct reflects the high flexibility of the proteins,^126^ and the presence of alpha-helices indicates the vaccine’s stability.^127^ Further refinement of the structure showed marked improvement, as noted in the negative Z-score and high amount of residues lying within the most favoured region, which indicates the model is of good quality.^128^ Validation of the vaccine construct structure using the Ramachandran plot and ProSA web server showed scores of 87.1% and −2.88, respectively. These values indicate the MEV is of quality owing that the values fall within the X-ray vicinity. The TLRs are vital in recognizing infectious agents and activating the pathway in the innate immune response. The TLR-2 is a cell surface protein highly conserved to identify molecules derived from micro-organisms known as PAMPs. It is important to note that TLR-2 is found in immune cells, epithelial cells, and fibrous tissue such as fibroblast. The TLR-2 also facilitates regulating various immune systems through balancing Th1 and Th2 immune responses.^129,130^ Surface-anchored antigens such as GPI act as ligands easily recognized by TLR-2 heterodimers of the host immune system. These functions of TLR-2 help reduce parasitic load, activate cytokine production, and switch antibody isotype.^131,132^ Data on TLR-2 characterization, identification, and cloning was first published in 1998.^133^ Since its discovery several decades ago, its importance in vertebrate immunity has been demonstrated in various studies.^73^ The TLR-2 ligands are mainly characterized by lipoproteins found in all bacteria with high expression in Gram-positive bacteria. They have a unique three-lipid (Triacyl) chain except for mycobacteria, which have two-lipid chain (diacyl).^134^ An essential feature of this ligand is the presence of a hydrophobic pocket that interacts with lipid chains, which allow variation in the length and chemical structure of lipids.^135^ In parasitic infection, GPI is activated by TLR-2. The response threshold is directly proportional to carbohydrate content and GPI lipid.^136^

All these factors enumerated earlier influenced the choice of TLR-2 and its success rate in previous immunoinformatics studies.^137,138^ Docking the MEV with TLR-2 and epitope docking (CD8 and CD4) to HLA alleles was done to analyse the binding interaction. This is an important step to identify the binding interaction of the MEV to the receptor and its ability to stimulate a sufficient immune response against babesiosis. The analysis showed that the highest binding energy had a significant affinity to the receptor and potentiated immune response. Molecular dynamic simulation of the vaccine revealed the stability and mobility of the vaccine construct. The results proved the MEV construct to be stable. The MDS was used to analyse the protein and ligand interactions at the atomic level.^139^ The RMSD and RMSF values indicate the complex’s stability after the simulation process. Despite the positive results of the simulation process, further physiological validation through experimental studies is required because MDS emulates atomistic molecular behaviour through water models and force fields.^140,141^ The MM/GBSA estimates the free energy of the binding of small ligands to the biological macromolecules.^142^ In this research, it was exclusively to estimate the free energy of the binding of the designed vaccine construct and TLR-2. The results of the energy estimate showed a significant contribution from electrostatic energy, van der Waals forces and gas phase energy. These interactions play an essential role in the binding affinity. Electrostatic interaction comprises salt bridges and hydrogen ions,^143,144^ which may be involved in the contribution of electrostatic energy.

The C-IMMSIM server was used to access the immunogenic profile of the MEV. The analysis showed that a single dose of the vaccine increased antigens, further stimulating the production of antibodies required before its decline 5 days post-vaccination. The IgM is usually induced first in early infections and later replaced by IgG as the memory immunity stage appears. An increase in B-memory cells, T-cytotoxic (TC) cells, and T-helper (TH) cell response was observed after exposure to the antigen, confirming the efficacy of the MEV consistent with its immunogenicity. A directly proportional relationship exists between T-helper cells and cytokine interferon, which plays a major role in modulating most parasitic infections.

*In silico* cloning of MEV into *E coli* k12 vector strain and analysis of the GAI and GC content was done. The values of the GAI and GC fall within the range of optimal expression, which suggests that the values are suitable for *in vitro* expression studies. The CAI expresses codon usage bias, which correlates with positive gene expression.^145^ The GC content within 30% to 70% indicate high potential for good protein expression and reproducibility.^146^ Due to the quantum of data available in the post-genomic era, discovering potential vaccine candidates and therapeutic targets has been greatly accelerated using computational tools. These tools’ availability has played a significant role in analysing genomes and protein sequences, identifying immunogenic proteins across diverse organisms and mapping epitopes. These have led to the development of multi-epitope-based vaccines. However, it is vital to know that it is purely a computational approach or *in silico* approach where a hypothetical protein construct was developed with no experimental validation, as it was done in this study. This is the limitation of this research.

The future direction will involve the synthesis of the peptides and further validation of these findings in pre-clinical and clinical trials under experimental conditions.

## Conclusions

Effectively utilizing computational tools has mitigated the challenges associated with conventional vaccine development. *In silico* analysis has expanded the scope of innovative medical research through novel research output, which will continue to shape the future of research. Using this approach, a highly selective structure was designed, which is immunogenic, antigenic, and non-allergenic, with a positive immune response after immune simulation. The MEV against babesiosis comprises 11 CTL epitopes, 17 HTL, and 3 B-cell epitopes incorporated into an adjuvant with the effective utilization of linkers. The designed vaccine was analysed by immunoinformatics approach with success. Further validation must be required through *in vivo* and *in vitro* experimentation.

## Supplemental Material

sj-docx-1-bbi-10.1177_11779322241287114 – Supplemental material for Multi-epitope Based Peptide Vaccine Candidate Against Babesia Infection From Rhoptry-Associated Protein 1 (RAP-1) Antigen Using Immuno-Informatics: An In Silico Approach Supplemental material, sj-docx-1-bbi-10.1177_11779322241287114 for Multi-epitope Based Peptide Vaccine Candidate Against Babesia Infection From Rhoptry-Associated Protein 1 (RAP-1) Antigen Using Immuno-Informatics: An In Silico Approach by Samson Anjikwi Malgwi, Victoria T Adeleke, Matthew Adekunle Adeleke and Moses Okpeku in Bioinformatics and Biology Insights
