# Supplementary material for: Multi-epitope Based Peptide Vaccine Candidate Against Babesia Infection From Rhoptry-Associated Protein 1 (RAP-1) Antigen Using Immuno-Informatics: An In Silico Approach
Source: Bioinform Biol Insights. 2024 Dec 16;18:11779322241287114. doi: 10.1177/11779322241287114 (PMC11650595; doi:10.1177/11779322241287114)
Supplement: sj-docx-1-bbi-10.1177_11779322241287114 – Supplemental material for Multi-epitope Based Peptide Vaccine Candidate Against Babesia Infection From Rhoptry-Associated Protein 1 (RAP-1) Antigen Using Immuno-Informatics: An In Silico Approach [file sj-docx-1-bbi-10.1177_11779322241287114.docx]

**Supplementary Table 1.** Accession numbers and species of *Babesia* parasites obtained from NCBI database

| S/No | Accession Number | Parasites |
| --- | --- | --- |
| 1. | >AGU67932.1 | *Babesia bovis* |
| 2. | >AGU67931.1 | *Babesia bovis* |
| 3. | >AGU67930.1 | *Babesia bovis* |
| 4. | >AGU67929.1 | *Babesia bovis* |
| 5. | >AGU67928.1 | *Babesia bovis* |
| 6. | >AGU67927.1 | *Babesia bovis* |
| 7. | >QQP60385.1 | *Babesia bovis* |
| 8. | >AHE41358.1 | *Babesia bigemina* |
| 9. | >AHE41357.1 | *Babesia bigemina* |
| 10. | >AHE41356.1 | *Babesia bigemina* |
| 11. | >AHE41355.1 | *Babesia bigemina* |
| 12. | >AHE41354.1 | *Babesia bigemina* |
| 13. | >AHE41353.1 | *Babesia bigemina* |
| 14. | >AHE41352.1 | *Babesia bigemina* |
| 15. | >AHE41351.1 | *Babesia bigemina* |
| 16. | >AHE41350.1 | *Babesia bigemina* |
| 17. | >AHE41349.1 | *Babesia bigemina* |
| 18. | >AHE41348.1 | *Babesia bigemina* |
| 19. | >AHE41347.1 | *Babesia bigemina* |
| 20. | >AHE41346.1 | *Babesia bigemina* |
| 21. | >AFP32917.1 | *Babesia bovis* |
| 22. | >BCO16762.1 | *Babesia bovis* |
| 23. | >BCO16761.1 | *Babesia bovis* |
| 24. | >BCO16760.1 | *Babesia bovis* |
| 25. | >BCO16759.1 | *Babesia bovis* |
| 26. | >BCO16758.1 | *Babesia bovis* |
| 27. | >BCO16757.1 | *Babesia bovis* |
| 28. | >BCO16756.1 | *Babesia bovis* |
| 29. | >BCO16755.1 | *Babesia bovis* |
| 30. | >BCO16754.1 | *Babesia bovis* |
| 31. | >AVM80888.1 | *Babesia bigemina* |
| 32. | >AVM80887.1 | *Babesia bigemina* |
| 33. | >AVM80886.1 | *Babesia bigemina* |
| 34. | >AVM80884.1 | *Babesia bigemina* |
| 35. | >AVM80882.1 | *Babesia bigemina* |
| 36. | >AVM80880.1 | *Babesia bigemina* |
| 37. | >AVM80879.1 | *Babesia bigemina* |
| 38. | >AMN09917.1 | *Babesia bigemina* |
| 39. | >AMN09916.1 | *Babesia bigemina* |
| 40. | >AMN09915.1 | *Babesia bigemina* |
| 41. | >AMN09914.1 | *Babesia bigemina* |
| 42. | >AMN09913.1 | *Babesia bigemina* |
| 43. | >AMN09912.1 | *Babesia bigemina* |
| 44. | >AEP32245.1 | *Babesia divergens* |
| 45. | >AEP32244.1 | *Babesia divergens* |
| 46. | >AEP32243.1 | *Babesia divergens* |
| 47. | >AEP32242.1 | *Babesia divergens* |
| 48. | >AEP32241.1 | *Babesia divergens* |
| 49. | >AEP32240.1 | *Babesia divergens* |
| 50. | >AEP32239.1 | *Babesia divergens* |
| 51. | >AEP32238.1 | *Babesia divergens* |
| 52. | >AEP32237.1 | *Babesia divergens* |
| 53. | >AEP32236.1 | *Babesia divergens* |
| 54. | >AEP32235.1 | *Babesia divergens* |
| 55. | >AEP32234.1 | *Babesia divergens* |
| 56. | >AVQ10355.1 | *Babesia bigemina* |
| 57. | >AIU41572.1 | *Babesia bovis* |
| 58. | >AIU41571.1 | *Babesia bigemina* |
| 59. | >AIU41570.1 | *Babesia bigemina* |
| 60. | >ALJ99833.1 | *Babesia bigemina* |
| 61. | >ALJ99832.1 | *Babesia bigemina* |
| 62. | >ALJ99831.1 | *Babesia begimina* |
| 63. | >ALJ99830.1 | *Babesia begimina* |
| 64. | >ALJ99829.1 | *Babesia bigemina* |
| 65. | >ALJ99828.1 | *Babesia bigemina* |
| 66. | >ALJ99827.1 | *Babesia bigemina* |
| 67. | >ALJ99826.1 | *Babesia bigemina* |
| 68. | >AFQ30755.1 | *Babesia bovis* |
| 69. | >AFQ30754.1 | *Babesia bovis* |
| 70. | >AFQ30753.1 | *Babesia bovis* |
| 71. | >AFY98852.1 | *Babesia bovis* |

**Supplementary Table 2:** The Population coverage analysis of the final multiepitope vaccine construct across the globe.

| Population/ Area | Population Coverage (%) | | |
| --- | --- | --- | --- |
|  | Class I (CD8+ T-cell) | Class II (CD4+ T-cell) | Class Combined |
| East Africa | 74.70 | 51.73 | 87.79 |
| Northeast Asia | 80.52 | 35.71 | 87.48 |
| South Asia | 78.35 | 60.15 | 91.37 |
| Southeast Asia | 67.43 | 34.14 | 78.55 |
| Southwest Asia | 73.12 | 30.91 | 81.43 |
| Europe | 90.73 | 69.34 | 97.16 |
| East Africa | 70.93 | 41.32 | 82.95 |
| West Africa | 81.94 | 40.01 | 89.17 |
| Central Africa | 65.56 | 32.49 | 76.75 |
| North Africa | 83.19 | 51.72 | 91.88 |
| South Africa | 73.93 | 0.00 | 73.93 |
| West Indies | 85.45 | 51.15 | 92.89 |
| North America | 87.63 | 68.22 | 96.07 |
| Central America | 2.19% | 21.22 | 22.94 |
| South America | 68.44 | 25.69 | 76.55 |
| Oceania | 61.16 | 44.31 | 78.37 |

**Supplementary Table 3**: The energy composition profile (Kcal/mol) based on MM/GBSA analysis of the TLR-vaccine complex

| **Energy component** | **Average** | **Standard error of mean** |
| --- | --- | --- |
| ΔE_VDW_ | -298.886 | 0.4185 |
| ΔE_EL_ | -3651.15 | 4.612 |
| ΔE_GB_ | 3849.071 | 4.665 |
| ΔE_SURF_ | -39.0174 | 0.0649 |
| ΔG_gas_ | -3950.04 | 4.8557 |
| ΔG_solv_ | 3810.053 | 4.615 |
| ΔG_bind_ | -139.982 | 0.3716 |

*E_VDW_* Van der Waals contribution, *E_EL_* Electrostatic energy, *E_GB_* Electrostatic contribution to the solvation of free energy, *E_SURF_* Non-polar solvation energy, ∆*G_gas_* gas phase energy, ∆*G_solvent_* solvation free energy, ∆*G_total_* final estimated binding free energy
